# What Underlies a Greater Reversal in Tactile Temporal Order Judgment When the Hands Are Crossed? A Structural MRI Study

**DOI:** 10.1093/texcom/tgab025

**Published:** 2021-04-05

**Authors:** Ali Moharramipour, Shigeru Kitazawa

**Affiliations:** Dynamic Brain Network Laboratory, Graduate School of Frontier Biosciences, Osaka University, Osaka 565-0871, Japan; Dynamic Brain Network Laboratory, Graduate School of Frontier Biosciences, Osaka University, Osaka 565-0871, Japan; Department of Brain Physiology, Graduate School of Medicine, Osaka University, Osaka 565-0871, Japan; Center for Information and Neural Networks, National Institute of Information and Communications Technology, Osaka University, Osaka 565-0871, Japan

**Keywords:** cortical characteristic, judgment reversal, spatial remapping, structural MRI, tactile temporal order judgment (TOJ)

## Abstract

Our subjective temporal order of two successive tactile stimuli, delivered one to each hand, is often inverted when our hands are crossed. However, there is great variability among different individuals. We addressed the question of why some show almost complete reversal, but others show little reversal. To this end, we obtained structural magnetic resonance imaging data from 42 participants who also participated in the tactile temporal order judgment (TOJ) task. We extracted the cortical thickness and the convoluted surface area as cortical characteristics in 68 regions. We found that the participants with a thinner, larger, and more convoluted cerebral cortex in 10 regions, including the right pars-orbitalis, right and left postcentral gyri, left precuneus, left superior parietal lobule, right middle temporal gyrus, left superior temporal gyrus, right cuneus, left supramarginal gyrus, and right rostral middle frontal gyrus, showed a smaller degree of judgment reversal. In light of major theoretical accounts, we suggest that cortical elaboration in the aforementioned regions improve the crossed-hand TOJ performance through better integration of the tactile stimuli with the correct spatial representations in the left parietal regions, better representation of spatial information in the postcentral gyrus, or improvement of top-down inhibitory control by the right pars-orbitalis.

## Introduction

If we are asked to judge the temporal order of two successive tactile stimuli delivered one to each hand, we often make more inverted judgments when our hands are crossed than when they are not crossed (Yamamoto and Kitazawa 2001; Shore et al. 2002). The finding has clearly shown that our brain cannot solely rely on the somatotopic locations of the stimuli in judging their temporal order. It is already 20 years from the initial reports, but there still remain controversies over two fundamental questions.

The first question is regarding why and how the inverted judgment occurs. Different theoretical accounts have been proposed to address this question (Kitazawa et al. 2008; Heed and Azanon 2014; Maij et al. 2020). Kitazawa et al. (2008) proposed that inverted judgment takes place because a stimulus to one hand in the crossed posture is initially mapped to the wrong hand in space, which will be remapped to the correct hand thereafter in 300–400 ms (Kitazawa 2002). This process of remapping from the wrong to the correct hand was initially proposed based on the time course of a curved somatosensory saccade (Groh and Sparks 1996) but was later confirmed by an ingenious psychological experiment (Azanon and Soto-Faraco 2008). Kitazawa et al. (2008) further proposed that the erroneous initial mappings are fixed as an inverted motion signal when two stimuli are successively delivered before the correct remapping is achieved, with a stimulus onset asynchrony (SOA) of 200 ms, for example. This inversion in the motion signal was suggested as a cause of an inverted judgment. Involvement of the motion signal was supported later by a functional imaging study (Takahashi et al. 2013). Another account was put forth by Shore et al. (2002). They proposed that the two external (spatial) and anatomical representations are concurrently available, thus, in the crossed posture, each hand will have two active left and right spatial characteristics. Integration of this contradictory spatial information may lead to associating incorrect hands with the tactile stimuli. Following this thread, Badde et al. proposed that the integration of the two representations is subject to top-down control as they found that the performance in tactile temporal order judgment (TOJ) task was modulated by the task instruction (Badde et al. 2016) and by a concurrent cognitive load (Badde et al. 2014). More recently, Maij et al. (2020) proposed that the TOJ performance does not rely on the external representation. They suggested that the external representation will be constructed post hoc on demand after making a hand choice, which depends on the different categorical features of touch, such as limb type and body side (Badde et al. 2019). In this account, inverted judgment occurs solely due to associating an incorrect hand with a tactile stimulus upon occurrence.

The second question concerns variabilities across participants. Due to crossing of the hands, some individuals show almost complete judgment reversal, but others show much less reversal (e.g., Yamamoto and Kitazawa 2001). The most evident factor reported thus far is the sex of participants. Generally, female participants show greater judgment reversal than male participants (Cadieux et al. 2010). However, there remains considerable diversity in the degree of judgment reversal even within male or female groups. In addition, the sex difference has been observed in only a few number of tactile TOJ studies (Unwalla et al. 2020). It is possible that sex is not a primary factor but that there exist some critical parameters in the cortical structure that explain the variability in both males and females.

In the present study, we aimed to search for such parameters in the cortical structure that would explain the inter-individual variability in the degree of judgment reversal. For this purpose, we examined the brain structure of 42 participants (23 males and 19 females) and extracted the cortical thickness and the surface and mean curvature of the white–gray matter boundary in 68 anatomical regions. Here, we show that a combined index of cortical features in a few regions explains the degree of judgment reversal regardless of the sex of the participants. Furthermore, we discuss the implications of the mechanisms of judgment reversal.

## Methods

### Participants

Forty-two volunteers (23 males and 19 females with an average age of 22.7 and a standard deviation [SD] of 2.1 years old) participated in this study. Thirty-nine of them were right-handed (laterality quotient between 70 and 100 with an average of $94.7$ and an SD of $7.3$), two of them were right-hand-preferred ambidextrous (laterality quotient of 10 and 15), and one of them was left-hand-preferred ambidextrous (laterality quotient of $-50$) according to the Edinburgh Inventory test (Oldfield 1971). All participants were neurologically normal and provided written informed consent according to the guidelines of the Ethical Review Board of Osaka University, Graduate School of Frontier Biosciences.

### Tactile TOJ Experiment

**Figure 1:**
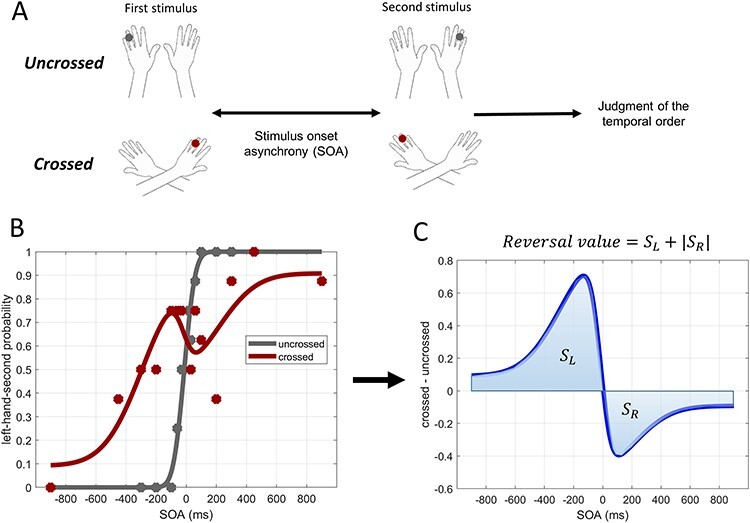
(*A*) Experimental paradigm. Two successive tactile stimuli were delivered one to the ring finger of each hand, and participants were asked to judge their temporal order by identifying which hand was stimulated second. The experiment was conducted in two sessions: In the first session, the hands were parallel (uncrossed), and in the second session, the hands were crossed. (*B*) Left-hand-second judgment probability of one particular participant in the crossed (red) and uncrossed (gray) conditions. SOA is the stimulus onset asynchrony (SOA) of the two tactile stimuli. Positive and negative SOAs indicate that the order of stimuli was from the right- to left-hand and from the left- to right-hand, respectively. (*C*) The difference between the judgment probabilities in the crossed and uncrossed conditions (blue curve) shows the judgment reversal that occurred due to the crossing of the hands. The surface area of judgment reversal was used as a measure to quantify the performance of the participants in the crossed-hand condition.

Participants sat behind a desk, with their head fixated on a chinrest, and placed their hands on the desk (palms facing down) with their arms either crossed or uncrossed (Fig. 1*A*). The distance between the ring fingers was kept at 20 cm in both of the postures. Participants closed their eyes and put on earphones that played white noise. They could only rely on their tactile sense. By using mechanical vibrators, two brief successive tactile stimuli were delivered to the ring finger of each hand. Participants were asked to judge the order of the stimuli by indicating which stimulus was delivered second. Two buttons were placed under the index finger of each hand. Participants expressed their judgment by pressing those buttons. Participants were instructed to respond as soon as they received the second stimulus (within 3 s). If they responded before the delivery of the second stimulus or after the 3 s response window, the trial was considered a miss, and it would be repeated again randomly at some point throughout the session. To prevent participants from making premature judgments based on the first stimulus, in some random trials, both stimuli were delivered to the same hand (catch trial). In the catch trials, participants were required to respond to the second stimulus as in the other trials. The catch trials were essential to make the participants pay attention to the order of the two stimuli. Otherwise, the participants could focus on the perception of the first stimulus in particular and then respond by the other hand without paying any attention to the second stimulus. We conducted the experiment in two sessions on the same day with a short break (5~10 min) in between. In the first session, the hands were parallel (uncrossed). In the second session, the hands were crossed.

The SOA of the two tactile stimuli was randomly assigned from 12 intervals ($\pm 15$, $\pm 30$, $\pm 60$, $\pm 100$, $\pm 200$, and $\pm 300$ ms) in the uncrossed session and from 14 intervals ($\pm 30$, $\pm 60$, $\pm 100$, $\pm 200$, $\pm 300$, $\pm 450$, and $\pm 900$ ms) in the crossed session. Negative and positive SOAs indicated that the order of stimuli was from the left-to-right hand and from right-to-left hand, respectively. Note that since the task is more difficult in the crossed session, longer SOAs were included to properly sample the entire spectrum of performance. In both of the sessions, the SOA of the catch trials was fixed at 100 ms. Each SOA interval was repeated eight times randomly. Therefore, the uncrossed session consisted of 112 trials (96 normal trials and 16 catch trials), and the crossed session consisted of 128 trials (112 normal trials and 16 catch trials). The inter-trial interval between the response (i.e., pressing the button) and the start of the next trial was randomly assigned a value between 500 and 1500 ms. Before each session, we conducted a short training session to familiarize the participants with the task.

To assess whether extracted metrics from the TOJ performances are reliably used as traits within individuals, we asked 24 of the participants (17 males and 7 females) to come again and repeat the TOJ task. Their second participation was scheduled between 3 and 12 months after their first participation. In the second participation, the conditions of the task were the same as the first participation except that the participants performed the crossed session first and then the uncrossed session. We reversed the order of sessions in the second participation to examine whether the order had any effect on the TOJ performances.

### Analysis of the TOJ Data

The probability of judging the left hand as the hand stimulated second (left-hand-second judgment probability) was calculated for each SOA in the crossed and uncrossed conditions. Figure 1*B* shows these probabilities (red and gray points) in one individual. It has been proposed that the judgment probability follows a sigmoid-shaped pattern (eq. 1) in the uncrossed condition and an *N*-shaped pattern (eq. 2) in the crossed condition (Yamamoto and Kitazawa 2001; Wada et al. 2004).

(1) \begin{equation*} {P}_u\left(\mathrm{SOA}\right)=\left({P}_{\mathrm{max}}-{P}_{\mathrm{min}}\right)\underset{-\infty }{\overset{\mathrm{SOA}}{\int }}\frac{1}{\sqrt{2\pi}{\sigma}_u}{\mathrm{e}}^{-{\left(\tau -{d}_u\right)}^2/2{\sigma_u}^2}\mathrm{d}\tau +{P}_{\mathrm{min}}. \end{equation*}

Equation (1) consists of one Gaussian cumulative distribution function that makes a sigmoid-shaped curve. ${P}_u$ is the left-hand-second judgment probability in the uncrossed condition, ${P}_{\mathrm{max}}$, ${P}_{\mathrm{min}}$, ${\sigma}_u$, and ${d}_u$ are the upper and lower asymptotes, the wideness, and the horizontal translation, respectively.

$${P}_c\left(\mathrm{SOA}\right)={f}_l\left(\mathrm{SOA}\right)\left[1-{P}_u\left(\mathrm{SOA}\right)\right]+\left[1-{f}_r\left(\mathrm{SOA}\right)\right]{P}_u\left(\mathrm{SOA}\right),$$

(2) \begin{equation*} \left\{\begin{array}{c}{f}_l\left(\mathrm{SOA}\right)={A}_l{\mathrm{e}}^{-{\left(\mathrm{SOA}-d\right)}^2/2{\sigma_f}^2}+c\\{}{f}_r\left(\mathrm{SOA}\right)={A}_r{\mathrm{e}}^{-{\left(\mathrm{SOA}-d\right)}^2/2{\sigma_f}^2}+c\end{array}\right.\kern-6pt. \end{equation*}

Equation (2) consists of two up and down Gaussian flips (${f}_l$ and ${f}_r$), one on each side of the *y*-axis, making an *N*-shaped curve. ${P}_c$ is the left-hand-second judgment probability in the crossed condition; ${A}_l$ and ${A}_r$ are the peaks of the up and down flips, respectively; and ${\sigma}_f$, $d$, and $c$ are the width, the horizontal translation, and the vertical translation of the flips, respectively. The variables in equations (1) and (2) were calculated by using the maximum likelihood estimation and the MATLAB optimization toolbox. Figure 1*B* shows the fitted curves in one individual. Note that the purpose of these curve fittings is to make the overall evaluation of the participant’s performance more robust and reliable. The model was not rejected in 38 out of the 42 participants by the goodness of fit test using the Pearson’s chi-square statistic $(P>0.05,{\chi}^2<15.5$, $\mathrm{d}f=8$ in the crossed condition and ${\chi}^2<14.1$, $\mathrm{d}f=7$ in the uncrossed condition) (Linhart and Zucchini 1986). In the four cases in which data did not pass the goodness of fit test, we observed the data by the eye. We judged that the model captured the essential characteristics of the data because the determination coefficient was greater than 0.4 (Supplementary Fig. S1).

By subtracting the left-hand-second judgment probability curve of the crossed condition from the uncrossed condition (Fig. 1*C*), we can observe the judgment reversal (i.e., erroneous judgments) that occurred due to the crossing of the hands. The absolute surface area of this subtracted curve was used as a measure to quantify the degree of judgment reversal. We called this measure the reversal value. We normalized the reversal value by dividing it by its maximum possible value. The normalized reversal value ranged between 0 and 1, with 0 indicating absolutely no judgment reversal and 1 indicating a complete judgment reversal over all of the SOAs. To test which aspects of the flip model were reflected in the reversal value, we examined the correlations between the reversal value and the model parameters in equation (2) (${A}_l$, ${A}_r$, ${\sigma}_f$, and $c$).

### Structural magnetic resonance imaging Acquisition

T1-weighted structural magnetic resonance imaging (MRI) images were acquired using 3-Tesla MRI scanners. Twelve of the participants were scanned with a MAGNETOM Vida (MP-RAGE sequence), and the other 30 were scanned with a MAGNETOM Prisma, Siemens scanner (gradient-recalled echo/inversion recovery sequence). Note that the resolution of the MRI images acquired from the two scanners was the same (slice thickness $=$ 1 mm, time repetition [TR] $=$ 1900 ms, time echo [TE] $=$ 3.37 ms, flip angle [FA] $=$ 9°, field of view [FOV] $=$ 256$\times$256 mm, and voxel size $=$ 1$\times$1$\times$1 mm).

One of the participants was scanned by both scanners. The participant was scanned first with the Vida scanner, and after 3 months, with the Prisma scanner. We compared the data of the two scanners to check whether there was any scanner bias affecting our analyses.

### Analysis of the Structural MRI Images

We used FreeSurfer software to analyze the structural MRI images (Fischl 2012). FreeSurfer finds the white–gray matter boundary and makes its 3D surface model using triangular meshes (approximately, 150 000 vertices per hemisphere) (Dale et al. 1999). The meshes of triangles allow us to measure the surface area, curvature, and cortical thickness at each vertex. The surface area of a vertex is the sum of the areas of its surrounding triangles divided by three. The cortical thickness of a vertex indicates its distance to its corresponding closest point on the pial–cerebrospinal fluid surface. Each vertex has two principal curvatures (${k}_1$ and ${k}_2$), which measure the maximum and minimum bending. The average value of ${k}_1$ and ${k}_2$ is known as the mean curvature ($H)$ (Pienaar et al. 2008; Ronan et al. 2011).

FreeSurfer automatically parcellates the cerebral cortex into 68 standard gyral-based anatomical regions (34 regions per hemisphere) known as the Desikan-Killiany atlas (Fischl et al. 2004; Desikan et al. 2006). We used those 68 regions to compare the structural properties of individual brains.

In choosing structural properties for the analyses, we paid particular attention to those that have been reported to correlate with intelligence in young participants as recruited in the present study (Luders et al. 2009; Schnack et al. 2015; Tadayon et al. 2020). We expected that such properties would be linked with cortical elaboration, or efficiency, which would have critical effects on the complex process of judgment reversal. It has been reported that cortical curvature and surface area correlated positively with intelligence, whereas cortical thickness correlated negatively (Luders et al. 2009; Schnack et al. 2015; Tadayon et al. 2020). This motivated us first to define a measure that combines the curvature and surface area that would reflect a kind of cortical efficiency or elaboration. The measure, termed rectified surface integral (RSI), was defined as follows:

(3) \begin{equation*} \mathrm{RSI}=\sum_A\left|H\right|\mathrm{d}A, \end{equation*}

where $H$ is the mean curvature, $\mathrm{d}A$ is the surface area, and $A$ refers to the vertices of one anatomical region. The RSI emphasizes the surface area ($\mathrm{d}A$) with a greater curvature ($H$) but ignores a flat surface. We expected that the RSI would provide a more reliable measure of cortical elaboration than a simple sum of the surface areas. To make the RSI robust to spatial distortions, we used a spatially smooth version of the mean curvature values produced by FreeSurfer in its analysis pipeline. We then defined the mean cortical thickness (MCT) by the following equation:

(4) \begin{equation*} \mathrm{MCT}=\frac{\sum_AT}{N_A}, \end{equation*}

where $T$ is the cortical thickness, and ${N}_A$ is the number of vertices of one anatomical region. We further introduced the ratio of RSI to MCT (RSI/MCT) in an expectation that it would serve as a more sensitive positive measure of cortical elaboration. We also examined the gray matter volume because a recent study reported that fluid intelligence was associated with the parameter (Chen et al. 2020).

We tested whether there was any scanner bias by comparing the data of the one participant who was scanned by both scanners. There was no scanner bias in the RSI, but there was a significant scanner bias in the MCT. On average, MCT was 2% smaller in the Vida than the Prisma scanner (Supplementary Fig. S2). This small difference was reasonable because the cortical thickness was reported to be sensitive to the scanner types (Iscan et al. 2015). We confirmed that a correction of the MCT by 2% in the 12 participants who were scanned by the Vida scanner did not alter the basic findings in the present study. As there was no bias in the RSI and the bias in the MCT was small (2%), the data of the Prisma and Vida scanners were analyzed together.

We examined the correlation between each of the cortical measures (i.e., MCT, RSI, and RSI/MCT) and the reversal value. As we had 68 anatomical regions and subsequently 68 correlation values, to find the significant regions, we corrected the *P* values for multiple (i.e., 68) tests by using the Benjamin–Hochberg false discovery rate (FDR) (Benjamini and Hochberg 1995). We repeated the same correlation analyses by using the mean of ${A}_l$ and ${A}_r$ (mean peak flip), which would reflect the pure degree of judgment reversal in TOJ instead of the reversal value.

Finally, we searched for a model that could effectively estimate the reversal value from the cortical characteristics.

(5) \begin{equation*} \mathrm{Reversal}\ \mathrm{value}\sim g\left({\sum}_{i=1}^N{\propto}_i{F}_i+C\right), \end{equation*}

(6) \begin{equation*} g(z)={\int}_{-\infty}^z\frac{1}{\sqrt{2\pi}}{\mathrm{e}}^{-{x}^2/2}\mathrm{d}x. \end{equation*}

The model was made up of a linear combination of the cortical characteristics (${F}_i$) passing through a normal cumulative distribution function ($g$) (eqs. 5 and 6, Fig. 6*A*). The normal cumulative distribution function was adopted to make the output of the model fall between 0 and 1 and within the range of the reversal value. Moreover, it can improve the model by accounting for a possibly existing nonlinearity between the cortical characteristics and the reversal value. By testing the model with and without the nonlinear layer (i.e., normal cdf), we realized that the nonlinearity of the model was beneficial. Basically, after identifying the cortical characteristics that had significant correlation with the reversal value, we tried all possible combinations of one, two, three, and up to eight of those cortical characteristics in the model. In addition to the cortical characteristics, we considered sex as a factor in the model to assess its possible significance. The Stan programming language (Carpenter et al. 2017) was used to fit the model to the data. We employed the leave-one-out (LOO) cross-validation (Vehtari et al. 2017) to find the most effective model (i.e., the combination of cortical characteristics that could effectively explain the reversal value). Moreover, we calculated Pareto *k*, an estimate of the distance between an individual LOO distribution and the full distribution. A Pareto *k* greater than 0.7 suggests that the left-out data were an outlier and that the model was not adequate. The model with the lowest LOO information criterion (LOOIC) and with all 42 Pareto *k* values smaller than 0.7 was selected as the best model (Fig. 6*B*).

**Figure 2:**
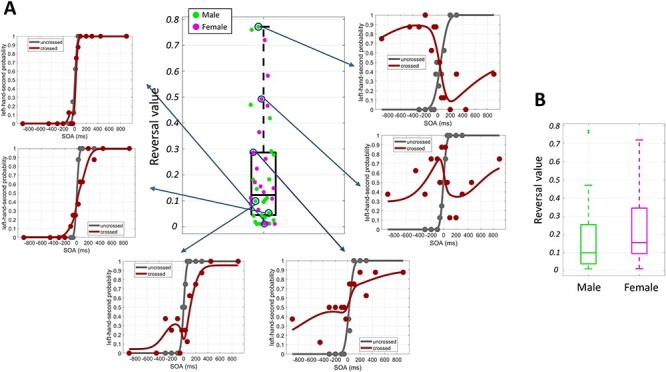
(*A*) Reversal values of the participants. Reversal values range between 0 and 1. Zero indicates absolutely no reversal, and 1 indicates complete reversal. A lower reversal value corresponds to a better performance in the crossed-hand tactile TOJ task (Fig. 1*A*). The judgment probabilities of the crossed and uncrossed conditions are shown for some reversal values. When the reversal value is lower, the difference between the crossed and uncrossed conditions is smaller. Male and female participants are shown with green and magenta colors, respectively. (*B*) Reversal values of the male and female participants. On average, females showed a greater reversal value than males; however, the difference was not significant (Wilcoxon rank-sum test, *P* value = 0.23).

### Data/Code Availability

The data that support the findings of this study are openly available at http://dx.doi.org/10.17632/tfkdrc4yfw.1.

## Results

In the uncrossed condition, the participants generally responded correctly when the SOA was greater than 100 ms (e.g., gray sigmoid curve in Fig. 1*B*). By contrast, in the crossed condition, the participants often showed inverted judgment even with SOAs greater than 200 ms (e.g., *N*-shaped response curve in Fig. 1*B*). However, it is noteworthy that the degree of judgment reversal varied considerably across the participants (Fig. 2*A*). Some participants showed nearly complete reversal (e.g., inverted sigmoid, top right panel in Fig. 2*A*) or *N*-shaped response curves (e.g., right middle panel in Fig. 2*A*), but others made much fewer inverted judgments (e.g., left top and left middle panels in Fig. 2*A*). The female participants generally showed a larger reversal value than the male participants (Fig. 2*B*), but the difference was not significant (Wilcoxon rank-sum test, *P* value = 0.23).

**Figure 3:**
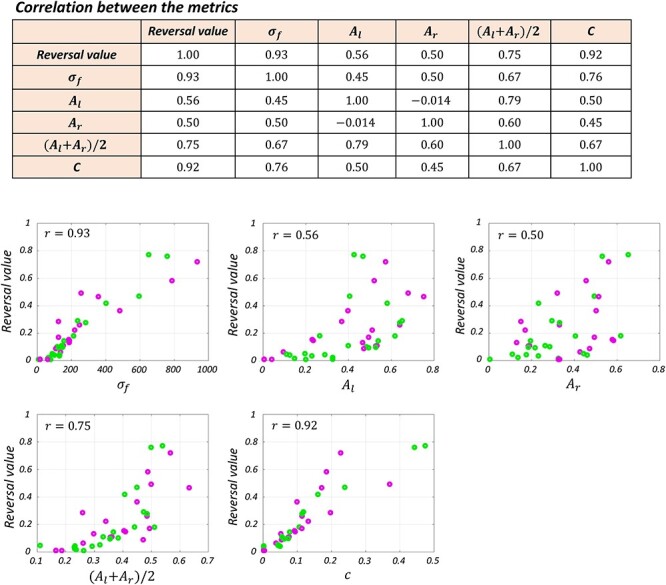
Correlation between the parameters of the Gaussian flip model (eq. 2) and the reversal value. The profile of the reversal value versus each of the parameters is shown in the subplots. Male and female participants are shown with green and magenta colors, respectively.

To summarize the crossing effect in the tactile TOJ task, we introduced the reversal value (Fig. 1*C*) which takes a value between 0 (no reversal) and 1 (full reversal). We confirmed that the reversal value correlated significantly with most of the parameters in the flip model of equation (2) (Fig. 3). It correlated significantly with the peak probability of the flips (${A}_l:r=0.56$, ${A}_r:r=0.50,P<0.001$) and their mean [$({A}_l+{A}_r)/2:r=0.75,P<{10}^{-7}$], which reflect the peak probability of judgment reversal. It was also highly correlated with the affected time window $({\sigma}_f:r=0.93,P<{10}^{-18})$, and the general spatial error $(c:r=0.92,P<{10}^{-17})$. The reversal value could thus be regarded as a comprehensive measure of the crossing effect.

Data of those 24 participants who performed the task for the second time revealed that the TOJ performance was highly reproducible (Supplementary Fig. S3). The reversal values of their first and second participations were highly correlated with each other ($r=0.95)$. The other model parameters also showed significant correlations (0.60, 0.70, 0.86, and 0.95 for ${A}_l$, ${A}_r$, ${\sigma}_f$, and $c$). The results agree with a recent report that the crossed-hands effect was highly reproducible within each individual participant (Unwalla et al. 2020).

The order of the crossed and uncrossed sessions was reversed in the second participation. The order did not significantly affect judgment reversal in each individual because there was no significant difference between the parameters in the first and second participations (Wilcoxon signed ranked test, *P* > 0.05). Taken together, the reversal value could be regarded as a comprehensive and reliable measure of judgment reversal in each individual participant.

**Figure 4:**
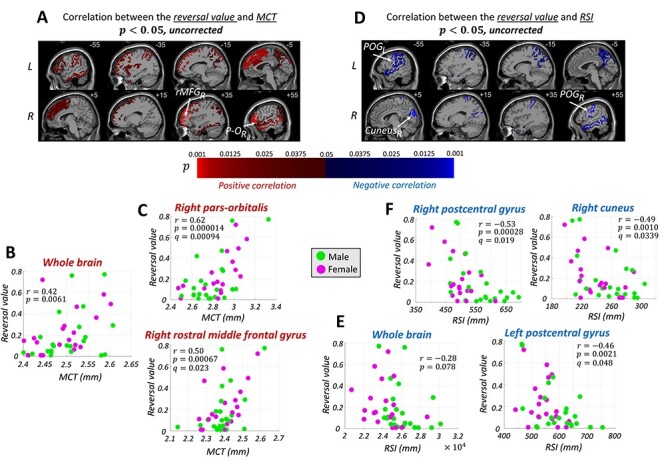
The brain anatomical regions (according to the Desikan-Killiany atlas) whose (*A*) MCT (*D*) RSI had a noticeable ($P<0.05$, uncorrected) correlation with the reversal value. Positive and negative correlations are shown with red and blue colors, respectively. The regions with a higher correlation value (i.e., lower *P* value) are shown with a brighter color. After correction for the false discovery of 0.05, in the MCT, right pars-orbitalis ($P-{O}_R$) and right rostral middle frontal gyrus $({r\mathrm{MFG}}_R),$ and in the RSI, right postcentral gyrus (${\mathrm{POG}}_R$), right cuneus (${\mathrm{cuneus}}_R$), and left postcentral gyrus (${\mathrm{POG}}_L$) survive as significant. The reversal value versus the (*B*) MCT of the whole brain and (*E*) RSI of the whole brain. The reversal value and (*C*) MCT (*F*) RSI profile of the regions that passed the FDR threshold of 0.05. $r$, $P$, and $q$ indicate the correlation value, *P* value and *q* value, respectively. Male and female participants are shown with green and magenta colors, respectively.

The MCT over the whole brain was positively correlated with the reversal value (Fig. 4*B*, $r=0.42,P=0.0061$). Region-by-region analyses (Fig. 4*A*, uncorrected *P* < 0.05) yielded many regions with positive correlations. After correction for the FDR of 0.05 (FDR correction), the two regions of the right pars-orbitalis (i.e., orbital part of the inferior frontal gyrus) and right rostral middle frontal gyrus were identified as significant. As shown in Fig. 4*C*, the MCT of these two regions had a strong positive correlation with the reversal value.

By contrast, the whole brain RSI was negatively correlated with the reversal value, though it did not reach the level of significance (Fig. 4*E*, $r=-0.28,P=0.078$). The region-by-region analyses (Fig. 4*D*, uncorrected, *P* < 0.05) yielded several regions with negative correlations. After the FDR correction, the right and left postcentral gyri and the right cuneus were identified as significant (Fig. 4*F*).

**Figure 5:**
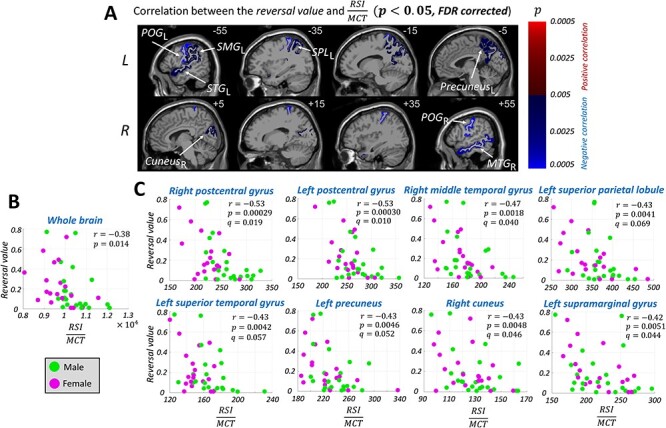
(*A*) The brain anatomical regions (according to the Desikan-Killiany atlas) whose RSI to MCT ratio had a significant ($P$ < 0.05, FDR-corrected) correlation with the reversal value. Positive and negative correlations are shown with red and blue colors, respectively. The regions with a higher correlation value (i.e., lower *P* value) are shown with a brighter color. (*B*) The reversal value versus the RSI to MCT ratio of the whole brain. (*C*) The RSI to MCT ratio and the reversal value profile of the regions with a significant correlation (i.e., right postcentral gyrus [${\mathrm{POG}}_R$], left postcentral gyrus [${\mathrm{POG}}_L$], right middle temporal gyrus [${\mathrm{MTG}}_R$], left superior parietal lobule [${\mathrm{SPL}}_L$], left superior temporal gyrus $[{\mathrm{STG}}_L$], left precuneus [${\mathrm{precuneus}}_L$], right cuneus [${\mathrm{cuneus}}_R$], and left supramarginal gyrus [${\mathrm{SMG}}_L$]). $r$, $P$, and $q$ indicate the correlation value, *P* value, and *q* value, respectively. Male and female participants are shown with green and magenta colors, respectively.

As for the RSI to MCT ratio, the following eight regions were identified after the FDR correction: the right and left postcentral gyri, right middle temporal gyrus, left superior parietal lobule, left superior temporal gyrus, left precuneus, right cuneus, and left supramarginal gyrus (Fig. 5*C*).

As for the gray matter volume, the correlations were generally weak and negative, with the strongest ones at the right postcentral gyrus ($r=-0.38$, $P=0.013$), left superior temporal gyrus ($r=-0.33$, $P=0.035$), and right entorhinal cortex ($r=-0.31$, $P=0.042$). None of the 68 regions survived the FDR correction.

Table 1 summarizes the main findings of our analysis by listing the 10 brain regions that showed a significant correlation between either of the MCT, RSI, or RSI/MCT and the reversal value. When we applied the same analyses by using the mean peak flip ($({A}_l+{A}_r)/2$) instead of the reversal value, the major findings remained unchanged: 8 of the 10 regions showed significant correlation (Table 1, Supplementary Fig. S4).

**Figure 6:**
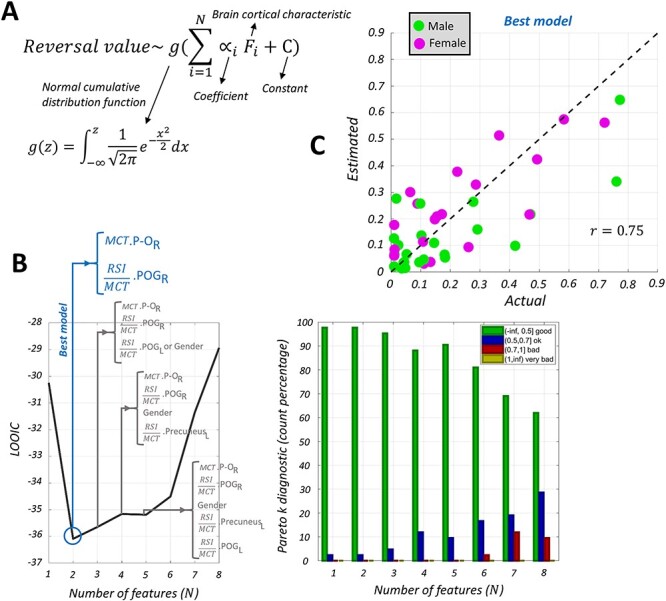
(*A*) Generalized linear model with a nonlinear sigmoid function (normal cumulative distribution function) that relates the cortical characteristics to the reversal value. (*B*) The LOO cross-validation approach to finding the best model. The lowest LOOIC is plotted against the number of features on the left, and the distributions of corresponding Pareto-k values are shown on the right. $P\hbox{-}{O}_R$: right pars-orbitalis, ${\mathrm{POG}}_R$: right postcentral gyrus, ${\mathrm{POG}}_L$: left postcentral gyrus. (*C*) Actual reversal values versus the reversal values estimated from the best model. Male and female participants are shown with green and magenta colors, respectively.

Last, we discovered that a model made up of the following two cortical features could most effectively explain the variability in judgment reversal (Fig. 6): 1) the MCT of the right pars-orbitalis and 2) the RSI to MCT ratio of the right postcentral gyrus. It is noteworthy that the addition of sex to the model did not improve the model in terms of the LOOIC (Fig. 6*B*). As shown in Fig. 6*C*, the model estimated the reversal value quite precisely ($r=0.75$) for both male and female participants.

**Table 1:** Summary of the correlation analysis

Brain region	Reversal value			Mean peak flip		
	MCT	RSI	RSI/MCT	MCT	RSI	RSI/MCT
Right pars-orbitalis	$\mathbf{0.62}$	$-0.064$	$-0.29$	$\mathbf{0.47}$	$-0.14$	$-0.31$
Right middle frontal gyrus	$\mathbf{0.50}$	$-0.23$	$-0.36$	$0.36$	$-0.31$	$-0.38$
Right postcentral gyrus	$0.20$	$-\mathbf{0.53}$	$-\mathbf{0.53}$	$-0.012$	$-\mathbf{0.51}$	$-\mathbf{0.44}$
Right cuneus	0.030	$-\mathbf{0.49}$	$-\mathbf{0.43}$	$0.0$ 64	$-0.40$	$-0.38$
Right middle temporal gyrus	$0.40$	$-0.42$	$-\mathbf{0.47}$	$0.38$	$-\mathbf{0.48}$	$-\mathbf{0.52}$
Left postcentral gyrus	$0.27$	**0.46**	$-\mathbf{0.53}$	$0.23$	$-0.42$	$-\mathbf{0.49}$
Left superior parietal lobule	$0.38$	$-0.34$	$-\mathbf{0.43}$	$\mathbf{0.48}$	$-0.39$	$-\mathbf{0.51}$
Left superior temporal gyrus	$0.18$	$-0.40$	$-\mathbf{0.43}$	$0.21$	$-\mathbf{0.47}$	$-\mathbf{0.51}$
Left precuneus	$0.31$	$-0.38$	$-\mathbf{0.43}$	$0.20$	$-0.42$	$-\mathbf{0.44}$
Left supramarginal gyrus	$0.36$	$-0.38$	$-\mathbf{0.42}$	$0.35$	$-\mathbf{0.46}$	$-\mathbf{0.50}$
Whole brain	$\mathbf{0.42}$	$-0.28$	$-\mathbf{0.38}$	$\mathbf{0.39}$	$-\mathbf{0.41}$	$-\mathbf{0.50}$

Note: Ten brain regions whose cortical measures (MCT, RSI, RSI/MCT) were significantly correlated (FDR corrected, *P* < 0.05), with the reversal value or the mean peak flip listed in the rows. Numbers in bold represent the correlation values that passed the FDR threshold of 0.05. RSI/MCT: RSI to MCT ratio.

## Discussion

In the current study, we examined the correlation between the structural MRI images of normal healthy individuals and their ability to perform a tactile TOJ task when their hands were crossed. We found that the cortical structural features (MCT, RSI, and RSI/MCT) of the following 10 regions significantly correlated with the individuals’ performance on the task: the right and left postcentral gyri, right middle temporal gyrus, left superior parietal lobule, left superior temporal gyrus, left precuneus, right cuneus, left supramarginal gyrus, right pars-orbitalis, and right rostral middle frontal gyrus (Table 1). Moreover, we discovered that knowing the cortical characteristics in just two of those regions, the right pars-orbitalis and right postcentral gyrus, was essentially adequate to effectively explain the variability in judgment reversal (Fig. 6). It is worth noting that the addition of sex did not significantly improve the model. When we directly compared the reversal value, the female participants showed a larger reversal value than the male participants on average, but the difference was not significant. These findings generally agree with a previous study that examined the sex effect in a large set of different tactile TOJ studies and reported significant sex effect (larger reversal in females than males) in only 3 out of 23 studies (Unwalla et al. 2020). Therefore, sex would not be a primary factor that affects the degree of judgment reversal, though it may serve as an additional factor. We discovered that, in general, the participants with a thinner (lower MCT), larger, and more convoluted (higher RSI) cerebral cortex in a few critical regions tended to have a smaller degree of judgment reversal in both males and females.

### Implications of the MCT and RSI

The MCT (thickness) correlated positively, but the RSI (convoluted surface area) correlated negatively with the reversal value. As a result, the gray matter volume, multiplication of the cortical thickness, and surface area did not show any significant correlation. It is worth discussing what a thinner, larger, and more convoluted cortex would mean regarding cortical functions, in general.

A higher cortical surface area means a higher number of cortical columns (Lübke and Feldmeyer 2007), which is associated with a reduction in the fraction of columnar interconnections (Ringo 1991). It has been proposed that a higher number of cortical columns and subsequently a smaller percentage of interconnectedness lead to higher information capacity and specialization by allowing limited interference and overlapping of information (Ringo 1991; Tadayon et al. 2020). The increase of surface area in a certain brain region would thus lead to an increase in the information capacity and more specialized functionalities carried out in that region.

As for the thickness of the cortex, we should pay careful attention to age. Cortical thickness increases in childhood (on average, until the age of 10), then goes through thinning during adolescence, more drastically in more intelligent children (Shaw et al. 2006; Shaw et al. 2008; Schnack et al. 2015). As far as the young adults, the age group of the present study, are concerned, a negative association between the cortical thickness and general intelligence (Schnack et al. 2015; Tadayon et al. 2020), visual creativity (Tian et al. 2018), and visual perceptual acuity (Song et al. 2015) has been reported. Therefore, in this age group, a thinner cortex is possibly associated with a better and more efficient function. However, it is noteworthy that in older age groups, a thinner cortex might not correspond to higher efficiency but to the atrophy of the brain, as it has been reported that a younger group (20~40 years old) with a higher cortical thickness had a higher common intelligence than an older group (e.g., 50~80 years old) with a lower cortical thickness (Salthouse et al. 2015).

### Roles of the 10 Regions in Crossed-Hand TOJ

We here discuss how the 10 regions listed in Table 1 can possibly influence one’s degree of judgment reversal. It is first worth noting that the majority of the regions have been implicated for the tactile TOJ by functional magnetic resonance imaging (fMRI) and magnetoencephalography (MEG) studies (Wada et al. 2012; Takahashi et al. 2013; Ora et al. 2016; Crollen et al. 2017; Takahashi and Kitazawa 2017).

In one fMRI study, Takahashi et al. (2013) investigated the neural correlates of tactile TOJ. They compared the TOJ task with the numerosity judgment (NJ) task, and in both tasks, the same set of tactile stimuli (braille pin stimuli) were delivered to both hands. The neural correlates of TOJ (TOJ > NJ) consisted of the right middle temporal gyrus, left supramarginal gyrus, left superior temporal gyrus, and right middle frontal gyrus. In another fMRI study, Crollen et al. (2017) found that the left precuneus, right middle temporal gyrus, and left superior parietal lobule were significantly activated during crossed-hand tactile TOJ compared with uncrossed-hand tactile TOJ (crossed > uncrossed). It has been found that the supramarginal gyrus plays a major role in the awareness and sense of hand position (Ben-Shabat et al. 2015; Findlater et al. 2018). It was reported in a lesion study that the superior parietal lobule plays a major role in updating and maintaining the internal representation of the body (Wolpert et al. 1998). Thus, more elaborate (more efficient) left superior parietal lobule and left supramarginal gyrus could be associated with more efficient remapping of tactile signals to the correct spatial locations (Kitazawa et al. 2008) or more efficient associations of tactile stimuli to their external representations (Shore et al. 2002). In agreement with the idea, Wada et al. (2012), in another fMRI study, found that adapting a crossed-hand posture activated the left superior temporal gyrus and the left posterior parietal areas (specifically, the left supramarginal gyrus when the eyes were closed), and the activity of the left posterior parietal areas was associated with the degree of judgment reversal. The same group, in a follow-up study, examined the left intraparietal-sulcus-seeded functional connectivity and reported significantly stronger functional connectivity in the right rostral–middle/inferior frontal gyrus and left posterior parietal areas during the crossed posture than during the uncrossed posture (Ora et al. 2016). Therefore, cortical elaboration in the right frontal regions (in addition to the left posterior parietal regions) might improve the process of updating the spatial coordinates of the hands.

On the other hand, the involvement of the left superior and the right middle temporal gyri, which involved so-to-speak biological motion areas, would be better understood by assuming the motion projection hypothesis put forth by Kitazawa and colleagues (Kitazawa et al. 2008; Takahashi et al. 2013; Takahashi and Kitazawa 2017). Cortical elaboration (efficiency) of these regions could be associated with a better function in replacing the initial erroneous motion signal with a correct one (Takahashi et al. 2013).

We have so far discussed the implications of 5 of the 10 regions (right middle frontal gyrus, right middle temporal gyrus, left superior parietal lobule, left superior temporal gyrus, and left supramarginal gyrus). Of the remaining five regions (the right pars-orbitalis, right and left postcentral gyri, left precuneus, and right cuneus), the left precuneus and the right cuneus were located in the medial part around the parieto-occipital sulcus. Takahashi and Kitazawa (2017), by recording MEG signals during crossed-hand tactile TOJ, found that judgment reversal was modulated by the α rhythm of the regions near the parieto-occipital sulcus. They hypothesized that the α rhythm regulates information flow from the superior colliculus, where the anatomical tactile information is represented, to the precuneus by modulating the thalamic nuclei that interconnect the superior colliculus to the precuneus at 10 Hz. Elaboration of the medial regions might thus contribute to better suppressing the anatomical tactile information coming from the subcortical regions, thereby reducing the chance of inverted judgments.

We finally turn to the postcentral gyrus and the right pars-orbitalis, both of which had a critical power to predict the reversal value but escaped from being identified by the previous imaging studies. We speculate that the activations of these regions in the tactile TOJ task were canceled by a similar amount of activations in the control tasks. As for the involvement of the postcentral gyrus, it is critically important to emphasize that the postcentral gyrus is more than just a simple primary sensory region and its most caudal part is especially responsible for the integration of bilateral somatosensory, proprioceptive, and visual information (Iwamura 1998; Borchers et al. 2011). Therefore, elaboration of the postcentral gyrus might contribute to the processing of not only somatotopic but also the spatial aspects of the tactile stimuli.

The pars-orbitalis is associated with top-down inhibitory control (Chavan et al. 2015; Yoo et al. 2016). It has been reported that the neural activation and morphological properties of the left and right pars-orbitalis were altered by improvements in a Go/No-Go task (Chavan et al. 2015). Moreover, it has been shown that symptom improvement in attention deficit hyperactivity disorder (ADHD) patients through psychological therapy significantly altered the resting-state regional homogeneity (i.e., local connectivity) of the right pars-orbitalis (Yoo et al. 2016). Thus, a more elaborate and efficient right pars-orbitalis might result in a better top-down control. This fits well with one of the accounts of the crossing effect that integration of external and anatomical spatial representations is regulated by top-down control (Badde et al. 2014; Badde et al. 2016; Badde and Heed 2016). Assuming this account, elaboration in the pars-orbitalis would improve a top-down control so that more weight is put on the external than the anatomical representations. Moreover, assuming the latest account which put forth that the inverted judgment occurs due to the association of the incorrect hand with the tactile stimulus upon occurrence (Maij et al. 2020), an improved top-down control is beneficial, as it would better inhibit the incorrect hand assignment.

In summary, elaboration of the regions listed in Table 1 can possibly improve one’s crossed-hand tactile TOJ performance through: 1) better integration of the tactile stimuli with the correct spatial representations in the left parietal regions, 2) improvement of motion signals in the motion areas, 3) suppressing the integration of the anatomical tactile signals of the superior colliculus in the precuneus, 4) better representation of spatial information in the postcentral gyrus, or 5) improvement of top-down inhibitory control by the right pars-orbitalis that expels the initial erroneous coupling between a tactile stimulus and the wrong hand. Future studies, designed to test each of these possibilities, would lead to a more comprehensive and elaborate model accounting for the crossing effect of the tactile TOJ task.

## Supplementary Material

SupplementaryFig1_tgab025

SupplementaryFig2_tgab025

SupplementaryFig3_tgab025

SupplementaryFig4_tgab025

## Notes

We thank Tamami Nakano, Chisa Ota, Marina Fukui, and Takuma Hashimoto for helping us to recruit participants and acquire their MRI images. *Conflict of Interest*: None declared.

## References

[ref1] Azanon E , Soto-Faraco S. 2008. Changing reference frames during the encoding of tactile events. Curr Biol. 18:1044–1049. 18619841 10.1016/j.cub.2008.06.045

[ref2] Badde S , Heed T. 2016. Towards explaining spatial touch perception: weighted integration of multiple location codes. Cogn Neuropsychol. 33:26–47. 27327353 10.1080/02643294.2016.1168791 PMC4975087

[ref3] Badde S , Heed T, Röder B. 2014. Processing load impairs coordinate integration for the localization of touch. Atten Percept Psychophys. 76:1136–1150. 24550040 10.3758/s13414-013-0590-2

[ref4] Badde S , Heed T, Röder B. 2016. Integration of anatomical and external response mappings explains crossing effects in tactile localization: a probabilistic modeling approach. Psychon Bull Rev. 23:387–404. 26350763 10.3758/s13423-015-0918-0

[ref5] Badde S , Röder B, Heed T. 2019. Feeling a touch to the hand on the foot. Curr Biol. 29:1491–1497.e1494. 30955931 10.1016/j.cub.2019.02.060

[ref6] Ben-Shabat E , Matyas TA, Pell GS, Brodtmann A, Carey LM. 2015. The right supramarginal gyrus is important for proprioception in healthy and stroke-affected participants: a functional MRI study. Front Neurol. 6:248. 26696951 10.3389/fneur.2015.00248 PMC4668288

[ref7] Benjamini Y , Hochberg Y. 1995. Controlling the false discovery rate: a practical and powerful approach to multiple testing. J R Stat Soc Ser B Stat Methodol. 57:289–300.

[ref8] Borchers S , Hauser T-K, Himmelbach M. 2011. Bilateral hand representations in human primary proprioceptive areas. Neuropsychologia. 49:3383–3391. 21864551 10.1016/j.neuropsychologia.2011.08.013

[ref9] Cadieux ML , Barnett-Cowan M, Shore DI. 2010. Crossing the hands is more confusing for females than males. Exp Brain Res. 204:431–446. 20574689 10.1007/s00221-010-2268-5

[ref10] Carpenter B , Gelman A, Hoffman MD, Lee D, Goodrich B, Betancourt M, Brubaker M, Guo J, Li P, Riddell A. 2017. Stan: a probabilistic programming language. J Stat Softw. 76:1–32. 10.18637/jss.v076.i01 PMC9788645 36568334

[ref11] Chavan CF , Mouthon M, Draganski B, Van Der Zwaag W, Spierer L. 2015. Differential patterns of functional and structural plasticity within and between inferior frontal gyri support training-induced improvements in inhibitory control proficiency. Hum Brain Mapp. 36:2527–2543. 25801718 10.1002/hbm.22789 PMC6869523

[ref12] Chen PY , Chen CL, Hsu YC, Cam CAN, Tseng WI. 2020. Fluid intelligence is associated with cortical volume and white matter tract integrity within multiple-demand system across adult lifespan. NeuroImage. 212:116576. 32105883 10.1016/j.neuroimage.2020.116576

[ref13] Crollen V , Lazzouni L, Rezk M, Bellemare A, Lepore F, Collignon O. 2017. Visual experience shapes the neural networks remapping touch into external space. J Neurosci. 37:10097–10103. 28947578 10.1523/JNEUROSCI.1213-17.2017 PMC6596542

[ref14] Dale AM , Fischl B, Sereno MI. 1999. Cortical surface-based analysis: I. segmentation and surface reconstruction. NeuroImage. 9:179–194. 9931268 10.1006/nimg.1998.0395

[ref15] Desikan RS , Ségonne F, Fischl B, Quinn BT, Dickerson BC, Blacker D, Buckner RL, Dale AM, Maguire RP, Hyman BT. 2006. An automated labeling system for subdividing the human cerebral cortex on MRI scans into gyral based regions of interest. NeuroImage. 31:968–980. 16530430 10.1016/j.neuroimage.2006.01.021

[ref16] Findlater SE , Hawe RL, Semrau JA, Kenzie JM, Amy YY, Scott SH, Dukelow SP. 2018. Lesion locations associated with persistent proprioceptive impairment in the upper limbs after stroke. NeuroImage: Clinical. 20:955–971. 30312939 10.1016/j.nicl.2018.10.003 PMC6180343

[ref17] Fischl B . 2012. FreeSurfer. NeuroImage. 62:774–781. 22248573 10.1016/j.neuroimage.2012.01.021 PMC3685476

[ref18] Fischl B , Van Der Kouwe A, Destrieux C, Halgren E, Ségonne F, Salat DH, Busa E, Seidman LJ, Goldstein J, Kennedy D. 2004. Automatically parcellating the human cerebral cortex. Cereb Cortex. 14:11–22. 14654453 10.1093/cercor/bhg087

[ref19] Groh JM , Sparks DL. 1996. Saccades to somatosensory targets. I. Behavioral characteristics. J Neurophysiol. 75:412–427. 8822567 10.1152/jn.1996.75.1.412

[ref20] Heed T , Azanon E. 2014. Using time to investigate space: a review of tactile temporal order judgments as a window onto spatial processing in touch. Front Psychol. 5:76. 24596561 10.3389/fpsyg.2014.00076 PMC3925972

[ref21] Iscan Z , Jin TB, Kendrick A, Szeglin B, Lu H, Trivedi M, Fava M, McGrath PJ, Weissman M, Kurian BT. 2015. Test–retest reliability of FreeSurfer measurements within and between sites: effects of visual approval process. Hum Brain Mapp. 36:3472–3485. 26033168 10.1002/hbm.22856 PMC4545736

[ref22] Iwamura Y . 1998. Hierarchical somatosensory processing. Curr Opin Neurobiol. 8:522–528. 9751655 10.1016/s0959-4388(98)80041-x

[ref23] Kitazawa S . 2002. Where conscious sensation takes place. Conscious Cogn. 11:475–477. 12435379 10.1016/s1053-8100(02)00031-4

[ref24] Kitazawa S , Moizumi S, Okuzumi A, Saito F, Shibuya S, Takahashi T, Wada M, Yamamoto S. 2008. Reversal of subjective temporal order due to sensory and motor integrations. In: Haggard P, Kawato M, Rossetti Y, editors. Attention and Performance XXII. Oxford: Oxford University Press, p. 73–97.

[ref25] Linhart H , Zucchini W. 1986. Model selection. New York, NY: John Wiley & Sons.

[ref26] Lübke J , Feldmeyer D. 2007. Excitatory signal flow and connectivity in a cortical column: focus on barrel cortex. Brain Struct Funct. 212:3–17. 17717695 10.1007/s00429-007-0144-2

[ref27] Luders E , Narr KL, Thompson PM, Toga AW. 2009. Neuroanatomical correlates of intelligence. Intelligence. 37:156–163. 20160919 10.1016/j.intell.2008.07.002 PMC2770698

[ref28] Maij F , Seegelke C, Medendorp WP, Heed T. 2020. External location of touch is constructed post-hoc based on limb choice. elife. 9:e57804. 32945257 10.7554/eLife.57804 PMC7561349

[ref29] Oldfield RC . 1971. The assessment and analysis of handedness: the Edinburgh inventory. Neuropsychologia. 9:97–113. 5146491 10.1016/0028-3932(71)90067-4

[ref30] Ora H , Wada M, Salat D, Kansaku K. 2016. Arm crossing updates brain functional connectivity of the left posterior parietal cortex. Sci Rep. 6:28105. 27302746 10.1038/srep28105 PMC4908406

[ref31] Pienaar R , Fischl B, Caviness V, Makris N, Grant PE. 2008. A methodology for analyzing curvature in the developing brain from preterm to adult. Int J Imaging Syst Technol. 18:42–68. 19936261 10.1002/ima.v18:1 PMC2779548

[ref32] Ringo JL . 1991. Neuronal interconnection as a function of brain size. Brain Behav Evol. 38:1–6. 1657274 10.1159/000114375

[ref33] Ronan L , Pienaar R, Williams G, Bullmore E, Crow TJ, Roberts N, Jones PB, Suckling J, Fletcher PC. 2011. Intrinsic curvature: a marker of millimeter-scale tangential cortico-cortical connectivity? Int J Neural Syst. 21:351–366. 21956929 10.1142/S0129065711002948 PMC3446200

[ref34] Salthouse TA , Habeck C, Razlighi Q, Barulli D, Gazes Y, Stern Y. 2015. Breadth and age-dependency of relations between cortical thickness and cognition. Neurobiol Aging. 36:3020–3028. 26356042 10.1016/j.neurobiolaging.2015.08.011 PMC4609615

[ref35] Schnack HG , Van Haren NE, Brouwer RM, Evans A, Durston S, Boomsma DI, Kahn RS, Hulshoff Pol HE. 2015. Changes in thickness and surface area of the human cortex and their relationship with intelligence. Cereb Cortex. 25:1608–1617. 24408955 10.1093/cercor/bht357

[ref36] Shaw P , Greenstein D, Lerch J, Clasen L, Lenroot R, Gogtay N, Evans A, Rapoport J, Giedd J. 2006. Intellectual ability and cortical development in children and adolescents. Nature. 440:676–679. 16572172 10.1038/nature04513

[ref37] Shaw P , Kabani NJ, Lerch JP, Eckstrand K, Lenroot R, Gogtay N, Greenstein D, Clasen L, Evans A, Rapoport JL, et al. 2008. Neurodevelopmental trajectories of the human cerebral cortex. J Neurosci. 28:3586–3594. 18385317 10.1523/JNEUROSCI.5309-07.2008 PMC6671079

[ref38] Shore DI , Spry E, Spence C. 2002. Confusing the mind by crossing the hands. Cogn Brain Res. 14:153–163. 10.1016/s0926-6410(02)00070-8 12063139

[ref39] Song C , Schwarzkopf DS, Kanai R, Rees G. 2015. Neural population tuning links visual cortical anatomy to human visual perception. Neuron. 85:641–656. 25619658 10.1016/j.neuron.2014.12.041 PMC4321887

[ref40] Tadayon E , Pascual-Leone A, Santarnecchi E. 2020. Differential contribution of cortical thickness, surface area, and gyrification to fluid and crystallized intelligence. Cereb Cortex. 30:215–225. 31329833 10.1093/cercor/bhz082 PMC7029693

[ref41] Takahashi T , Kansaku K, Wada M, Shibuya S, Kitazawa S. 2013. Neural correlates of tactile temporal-order judgment in humans: an fMRI study. Cereb Cortex. 23:1952–1964. 22761307 10.1093/cercor/bhs179

[ref42] Takahashi T , Kitazawa S. 2017. Modulation of illusory reversal in tactile temporal order by the phase of posterior alpha rhythm. J Neurosci. 37:5298–5308. 28450538 10.1523/JNEUROSCI.2899-15.2017 PMC6596459

[ref43] Tian F , Chen Q, Zhu W, Wang Y, Yang W, Zhu X, Tian X, Zhang Q, Cao G, Qiu J. 2018. The association between visual creativity and cortical thickness in healthy adults. Neurosci Lett. 683:104–110. 29936269 10.1016/j.neulet.2018.06.036

[ref44] Unwalla K , Kearney H, Shore DI. 2020. Reliability of the crossed-hands deficit in tactile temporal order judgements. Multisens Res. 1:1–35. 10.1163/22134808-bja10039 33706262

[ref45] Vehtari A , Gelman A, Gabry J. 2017. Practical Bayesian model evaluation using leave-one-out cross-validation and WAIC. Stat Comput. 27:1413–1432.

[ref46] Wada M , Takano K, Ikegami S, Ora H, Spence C, Kansaku K. 2012. Spatio-temporal updating in the left posterior parietal cortex. PLoS One. 7:e39800. 22768126 10.1371/journal.pone.0039800 PMC3387247

[ref47] Wada M , Yamamoto S, Kitazawa S. 2004. Effects of handedness on tactile temporal order judgment. Neuropsychologia. 42:1887–1895. 15381018 10.1016/j.neuropsychologia.2004.05.009

[ref48] Wolpert DM , Goodbody SJ, Husain M. 1998. Maintaining internal representations: the role of the human superior parietal lobe. Nat Neurosci. 1:529–533. 10196553 10.1038/2245

[ref49] Yamamoto S , Kitazawa S. 2001. Reversal of subjective temporal order due to arm crossing. Nat Neurosci. 4:759. 11426234 10.1038/89559

[ref50] Yoo JH , Oh Y, Jang B, Song J, Kim J, Kim S, Lee J, Shin H-Y, Kwon J-Y, Kim Y-H. 2016. The effects of equine-assisted activities and therapy on resting-state brain function in attention-deficit/hyperactivity disorder: a pilot study. Clin Psychopharmacol Neurosci. 14:357. 27776388 10.9758/cpn.2016.14.4.357 PMC5083948

