# Supplementary figures and images for: What Underlies a Greater Reversal in Tactile Temporal Order Judgment When the Hands Are Crossed? A Structural MRI Study
Source: Cereb Cortex Commun. 2021 Apr 5;2(2):tgab025. doi: 10.1093/texcom/tgab025 (PMC8152922; doi:10.1093/texcom/tgab025)

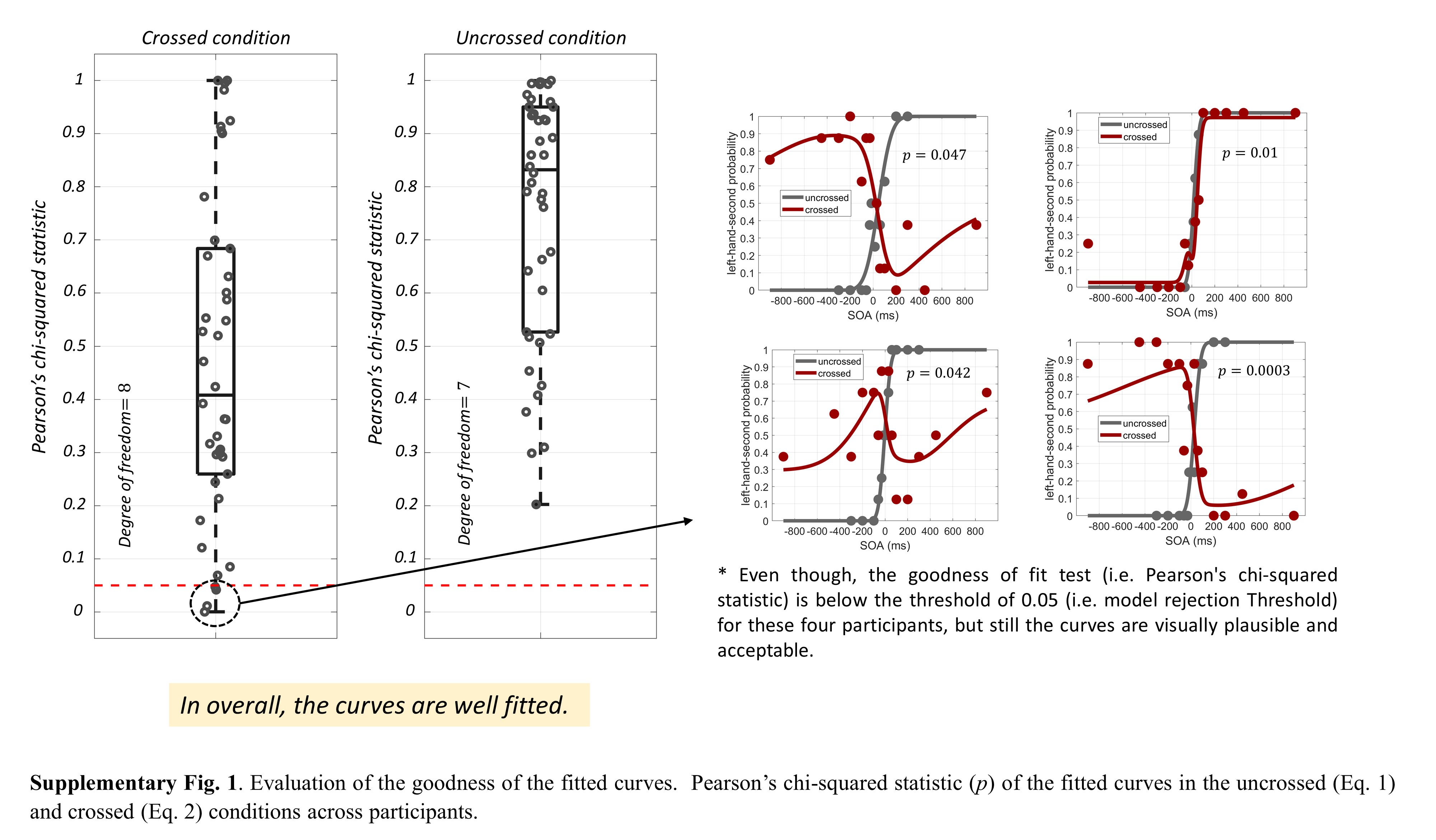

Supplement: SupplementaryFig1_tgab025 [file supplementaryfig1_tgab025.jpeg]

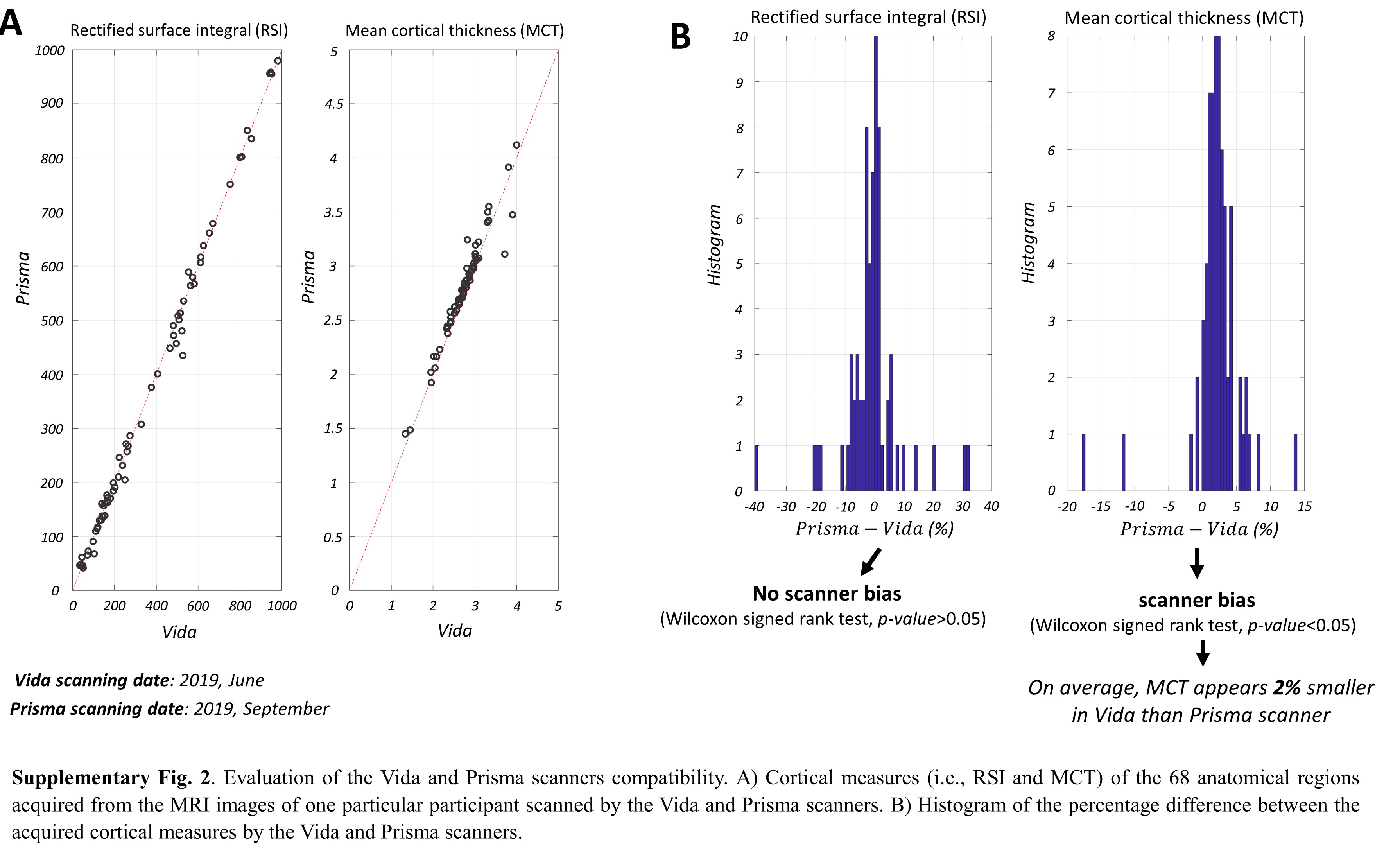

Supplement: SupplementaryFig2_tgab025 [file supplementaryfig2_tgab025.jpeg]

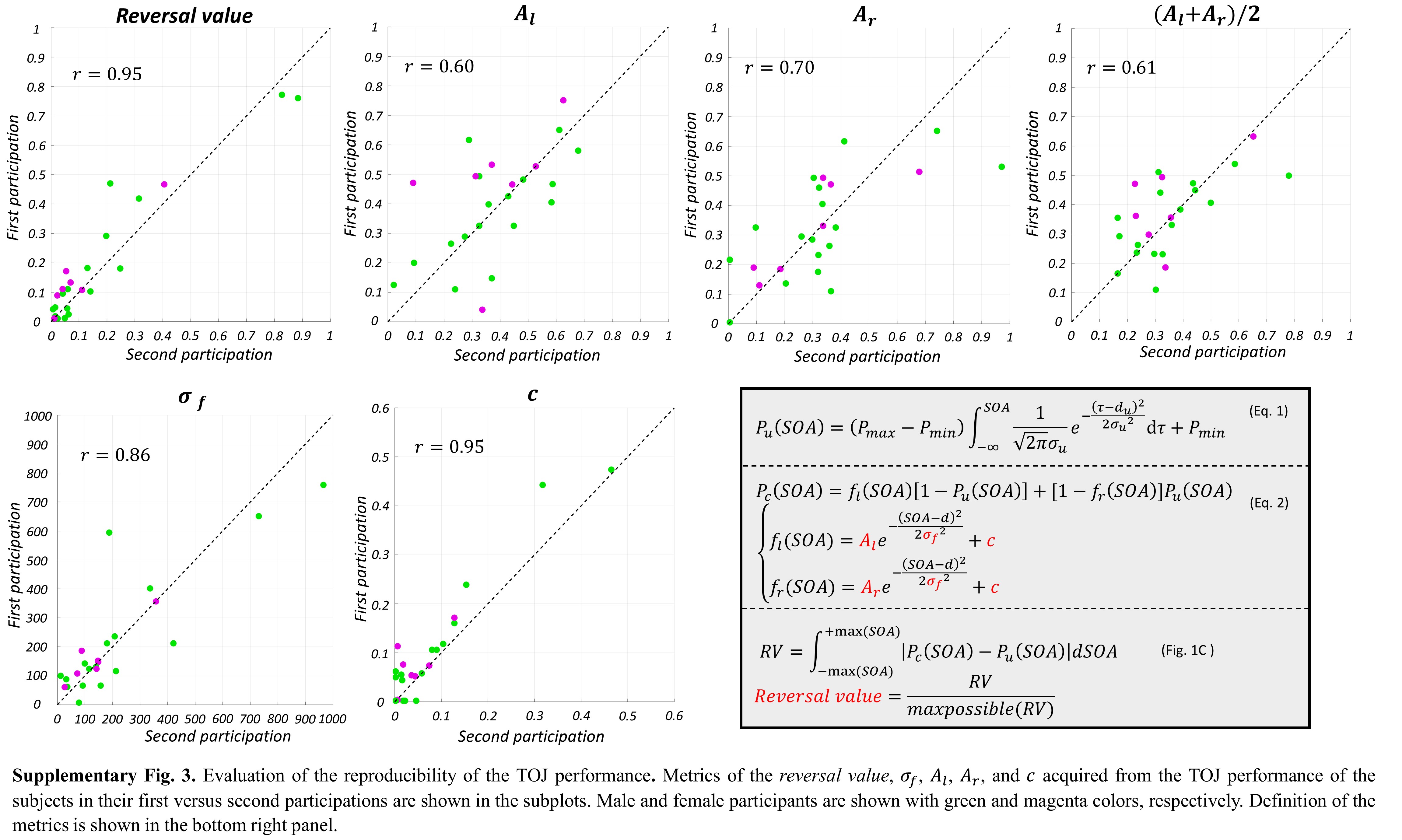

Supplement: SupplementaryFig3_tgab025 [file supplementaryfig3_tgab025.jpeg]

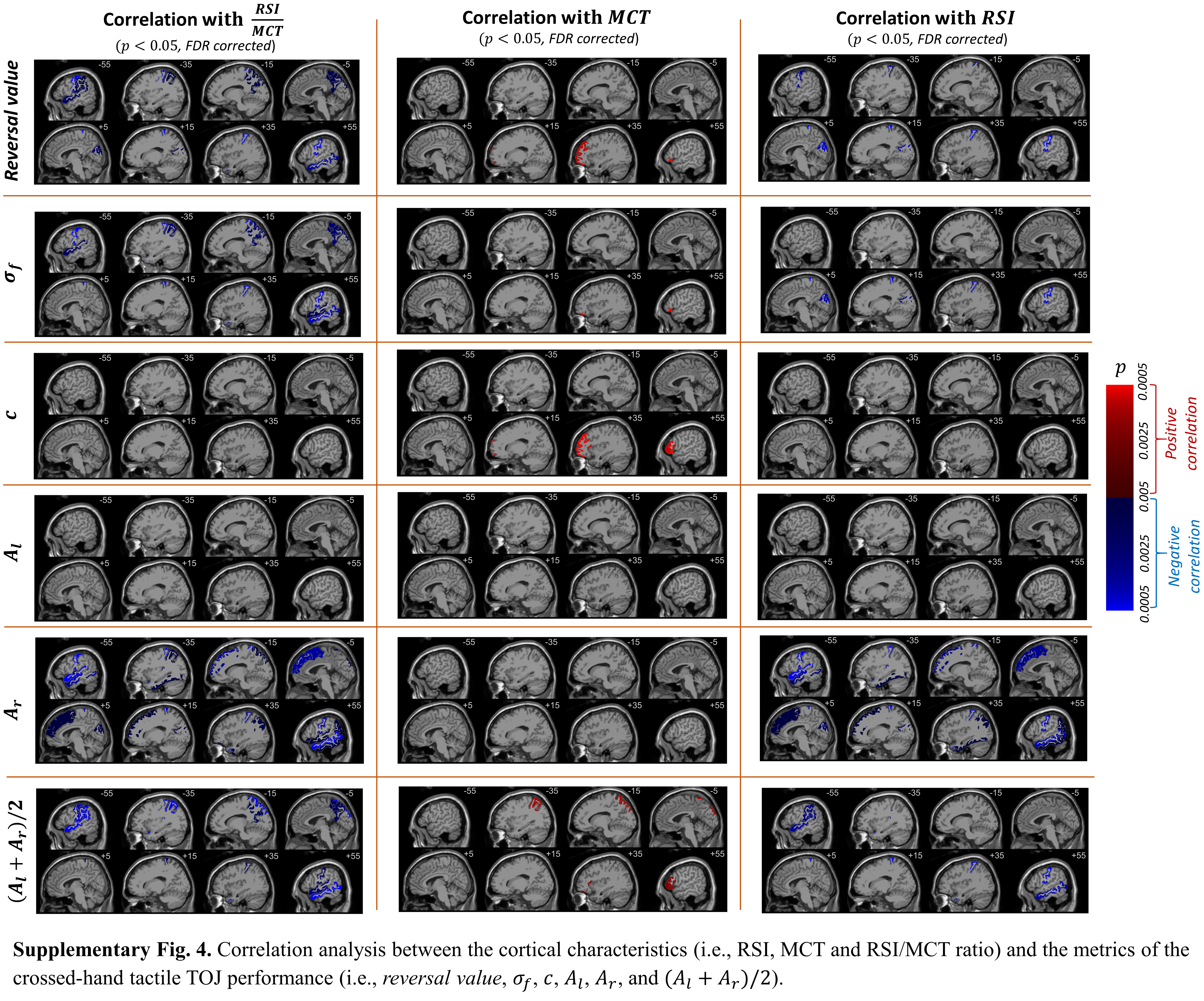

Supplement: SupplementaryFig4_tgab025 [file supplementaryfig4_tgab025.zip › SupplementaryFig4_tgab025.tif]
